# Experts’ memory superiority for domain-specific random material generalizes across fields of expertise: A meta-analysis

**DOI:** 10.3758/s13421-016-0663-2

**Published:** 2016-10-21

**Authors:** Giovanni Sala, Fernand Gobet

**Affiliations:** Department of Psychological Sciences, Bedford Street South, University of Liverpool, Liverpool, L69 7ZA UK

**Keywords:** Memory, Recall, Expertise, Random, Meta-analysis

## Abstract

Experts’ remarkable ability to recall meaningful domain-specific material is a classic result in cognitive psychology. Influential explanations for this ability have focused on the acquisition of high-level structures (e.g., schemata) or experts’ capability to process information holistically. However, research on chess players suggests that experts maintain some reliable memory advantage over novices when random stimuli (e.g., shuffled chess positions) are presented. This skill effect cannot be explained by theories emphasizing high-level memory structures or holistic processing of stimuli, because random material does not contain large structures nor wholes. By contrast, theories hypothesizing the presence of small memory structures—such as chunks—predict this outcome, because some chunks still occur by chance in the stimuli, even after randomization. The current meta-analysis assessed the correlation between level of expertise and recall of random material in diverse domains. The overall correlation was moderate but statistically significant ($\overline{r} = .41,p < .001$), and the effect was observed in nearly every study. This outcome suggests that experts partly base their superiority on a vaster amount of small memory structures, in addition to high-level structures or holistic processing.

A classic result in cognitive psychology is that experts have an excellent memory for meaningful material taken from their domain of expertise, even when this material is presented only briefly. This result was originally uncovered by De Groot’s (1965) and Chase and Simon’s (1973) study of chess players, and later replicated in many domains, including sports, science, engineering, and games (see Ericsson, Charness, Feltovich, & Hoffman, 2006; Gobet, 2015, for overviews). Experts’ superiority has often been explained by the acquisition of high-level knowledge structures (Chi, Feltovich, & Glaser, 1981; Cooke, Atlas, Lane, & Berger, 1993; Holding & Pfau, 1985; Kalyuga, Ayres, Chandler, & Sweller, 2003; Patel & Groen, 1986), or the ability to process information holistically, unlike nonexperts who have to process it piecemeal or analytically (Curby, Glazek, & Gauthier, 2009; Dreyfus & Dreyfus, 1986; Richler, Wong, & Gauthier, 2011). High-level knowledge structures, such as schemata and verbal concepts, abstract from the detail of the material to memorize. For example, in chess, a complex position could be summarized by the description “an Italian opening, variation Giuoco Pianissimo, with White’s pressure on the white squares.” With holistic processing, it is assumed that the scene or object being perceived is not decomposed into simpler units, but is processed as a unified whole.

In a meta-analysis of 13 studies, Gobet and Simon (1996b) showed that, at least with chess, these explanations were not sufficient to explain experts’ superiority. They found that experts maintained some superiority with random positions, in which any high-level structure had been destroyed. With such positions, experts’ advantage cannot be explained by the use of high-level structures (by construction, these do not exist in random positions) nor by holistic processing (there is no whole to process after the location of pieces has been randomized). Gobet and Simon’s (1996b) result was predicted by computer simulations based on the mechanism of chunking (Gobet & Simon, 1996a). As proposed by Chase and Simon (1973), expertise in chess is acquired by learning, through practice and study, a large number of chunks, which are units of both perception and meaning; in chess, chunks consist of constellations of pieces occurring often together in masters’ games. Experts’ superiority with meaningful material (game positions in chess) is explained by their ability to rapidly identify patterns present on the board, and retrieve chunks from their long-term memory (LTM). As shown by the computer simulations, some patterns still occur, by chance, in random positions; as experts are more likely to notice them due to their large store of chunks, they can maintain some superiority. Importantly, this superiority is not an artefact of the specific kind of randomization used, as proposed by Vicente and Wang (1998), because it is maintained with positions obtained with different methods of randomization (Gobet & Waters, 2003; Waters & Gobet, 2008).

Gobet and Simon’s (1996b) result is important theoretically, as it can readily be explained by theories based on chunking, such as chunking theory (Chase & Simon, 1973) and template theory (Gobet & Simon, 1996c), but not by theories focusing on high-level representations or holistic processing. However, it is unknown whether this result generalizes to other domains of expertise beyond chess. Therefore, the aim of this study was to establish whether experts maintain some memory superiority with random stimuli in different domains of expertise. Support for this hypothesis would strongly corroborate theories based on chunking.

## The present meta-analysis

The present meta-analysis aimed to evaluate two predictions of chunk-based theories on the recall of random material: (a) the positive correlation between expertise and performance in recalling domain-specific random material occurs regardless of the particular domain, and thus is not specific to chess, and (b) this skill effect is no more than moderate, because the number of meaningful chunks in unstructured material is heavily reduced after randomization. Thus, the skill effect is supposed to be relatively modest in size.

To test these two hypotheses, a systematic search of articles having used random material with experts and nonexperts was carried out, and an overall correlation expressing the relationship between expertise and the capacity of recalling random material was calculated. Then, a moderator analysis was run to evaluate whether the relationship between expertise and recall performance of random material was present in every domain. To evaluate the role of domain as moderator, the studies were categorized into five different domains: games, music, programming, sports, and others.

The prediction of chunk-based theories applies primarily with short presentation times, less than 8–10 seconds (time to create a new chunk in LTM; Gobet & Simon, 2000; Simon, 1969), where perceptual and short-term mechanisms dominate. As exposition time varies with the type of material to recall (e.g., seconds for game positions and music notes, minutes for computer programs), we also ran a moderator analysis to evaluate whether the exposition time affected the effect size. Exposition time is positively related to performance on the recall task with randomized chess positions (Gobet & Simon, 2000), but of course both novices and experts can take advantage of prolonged time to use alternative memory mechanisms (e.g., learning new chunks, semantics) and thus be able to recall more items.

Finally, because several studies reported only the direction of the effect (e.g., experts outperforming novices) without providing data sufficient to calculate an effect size, we also calculated—following the approach adopted in Gobet and Simon (1996b)—the probability of *k* occurrences of the skill effect out of the total number (*n*) of cases.

## Method

### Literature search

**Fig. 1:**
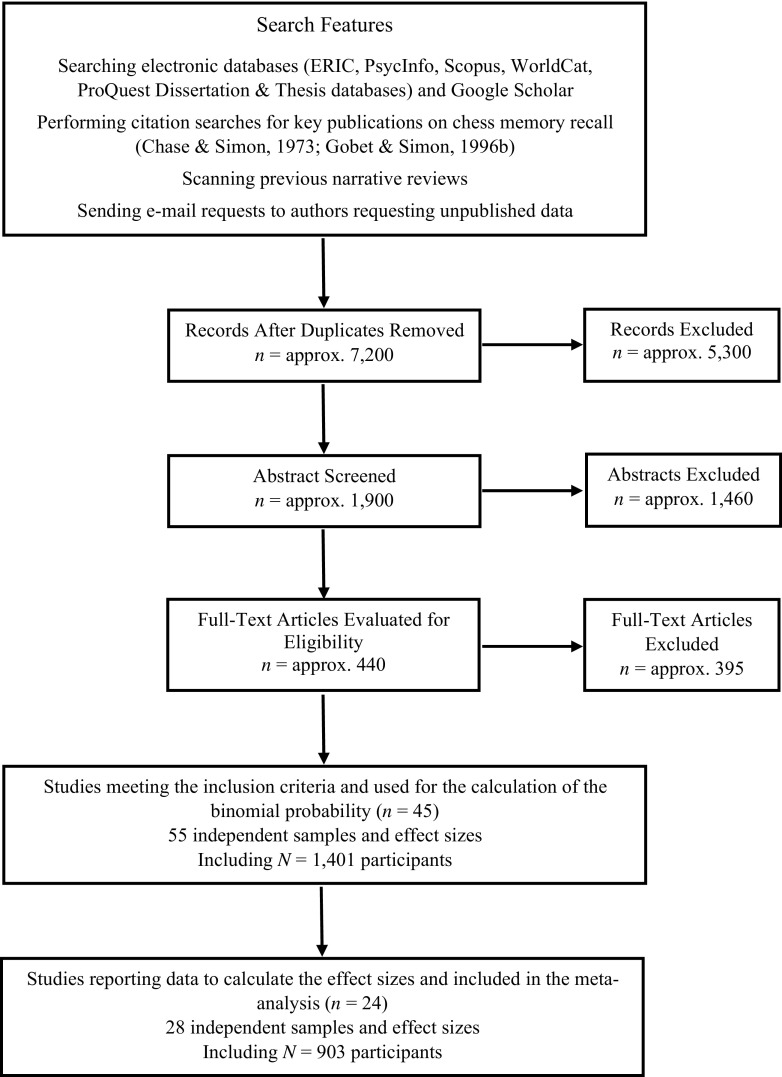
Flow diagram of the studies considered and ultimately included for the calculation of the binomial probability analysis and the meta-analysis

In line with the PRISMA statement (Moher, Liberati, Tetzlaff, & Altman, 2009), a systematic search strategy was used to find relevant studies (see Fig. 1 for a summary of the procedure). Using several combinations of the terms *recall, random, scrambled, unstructured, shuffled,* and *meaningless*, we searched ERIC, PsycInfo, Scopus, WorldCat, ProQuest Dissertation & Thesis databases, and Google Scholar to identify all the potential relevant studies. In addition, previous narrative reviews were examined, and we e-mailed researchers in the field (*n* = 13) asking them for unpublished studies and data. Finally, we performed citation searches for two publications: Chase and Simon (1973) and Gobet and Simon (1996b).

### Inclusion/exclusion criteria

The studies were included according to the following seven criteria:

1. The domain of expertise studied did not entail training memory per se; for example, memory experts using mnemonics (e.g., in the digit-span task), were excluded.
2. A measure of performance in a recall task was collected.
3. Some kind of random material was used.
4. The task was performed by participants with different levels of expertise (e.g., years of practice, categories, or Elo points).
5. Novices were not totally unfamiliar with the material to recall.^1^
6. The random material was obtained by shuffling *all* the elements (e.g., chess pieces, lines of programs) of structured material. No partially randomized material was considered.
7. The data presented were sufficient to establish the direction of the effect (e.g., experts better than novices) or, better, to calculate an effect size.

We found 45 studies conducted from January 1973 to March 2015 meeting the above criteria, including 1,401 participants, and 55 independent samples. These were included in a binomial distribution analysis. The 24 studies reporting sufficient data to calculate an effect size were included in a meta-analysis, and included 903 participants, 28 independent samples, and 28 effect sizes^2^ (see Table 1).

**Table 1:** Summary of the 28 samples included in the meta-analysis

Study	*N*	Domain	Description of the random material	Time of exposition to the stimulus (in seconds)^a^
Adelson (1981)	10	Programming	Lines of programs presented in a random order	20
Barfield (1986)	22	Programming	Lines of programs presented in a random order	300
Bateson, Alexander, and Murphy (1987)	50	Programming	Lines of programs presented in a random order	180
Charness (1979)	20	Games (Bridge)	Unstructured bridge hands	5
Chiesi, Spilich, and Voss (1979)	42	Sport (Baseball)	Random sentence presentation order of baseball events	not given
Engle and Bukstel (1978)	4	Games (Bridge)	Unstructured bridge hands	20
Gerard (1998)	100	Other (Diagrams)	Diagrams with labels randomized	180
Gobet and Simon (1996c)	13	Games (Chess)	Shuffled chess positions	5
Gobet and Simon (2000)	20	Games (Chess)	Shuffled chess positions	15
Gobet and Waters (2003)	36	Games (Chess)	Shuffled chess positions	5
Gong, Ericsson, and Moxley (2015)	23	Games (Chess)	Shuffled chess positions	5
Guerin and Matthews (1990)	104	Programming	Lines of programs presented in a random order	600
Holding and Reynolds (1982)	24	Games (Chess)	Shuffled chess positions	8
Kalakoski and Saariluoma (2001)	16	Other (Taxi drivers)	Random auditory presentation of streets	not given
Knecht (2003)	20	Music	Shuffled notes in a musical stave	not given
Magliaro and Burton (1986)	16	Programming	Lines of programs presented in a random order	120
Nakatani and Yamaguchi (2014)	24	Games (Shogi)	Shuffled shogi positions	5
Pezzulo, Borghi, Barca, and Bocconi (2010)	6	Sport (Climbing)	Impossible routes on a climbing wall	not given
Schmidt (1986)	20	Programming	Lines of programs presented in a random order	4
Schneider, Gruber, Gold, and Opwis (1993)—S1	40	Games (Chess)	Shuffled chess positions	10
Schneider, Gruber, Gold, and Opwis (1993)—S2	40	Games (Chess)	Shuffled chess positions	10
Schultetus and Charness (1999)	17	Games (Chess)	Shuffled chess positions	8
Sloboda, (1976)—S1	8	Music	Shuffled notes in a musical stave	2
Sloboda, (1976)—S2	8	Music	Shuffled notes in a musical stave	0.075
Sloboda, (1976)—S3	10	Music	Shuffled notes in a musical stave	2
Sloboda, (1976)—S4	10	Music	Shuffled notes in a musical stave	2
Weber and Brewer (2003)	48	Sport (Hockey)	Shuffled sentences of play from hockey matches	50.6
Zhilin and Tkachuk (2013)	152	Other (Chemistry)	Random sequences of chemical symbols	30

^a^ When the study presented only the overall performance of several trials, the mean time of exposition was reported in the table

### Effect sizes

As a measure of effect size, we used the correlation between expertise in a domain and performance in recalling random material. Two studies reported a correlation coefficient, which we used. When group-level comparisons (e.g., novices vs. experts) were reported (*k* = 26), we converted Cohen’s *d*s^3^ to point biserial correlation (Schmidt & Hunter, 2015). Artificial dichotomization was corrected for the effect sizes extrapolated from group-level comparisons only in chess studies, because only the field of chess—among the ones considered in the present meta-analysis—has a continuous variable assessing expertise (Elo, 1978).

### Moderators

The two potential moderators were as follows:

1. Domain (categorical variable): This variable includes games, music, programming, sports and others.
2. Time of exposition (dichotomous variable): The time of exposition (in seconds) to the material to recall was more than 8 seconds or less or equal than 8 seconds.

## Results

### Meta-analysis

**Fig. 2:**
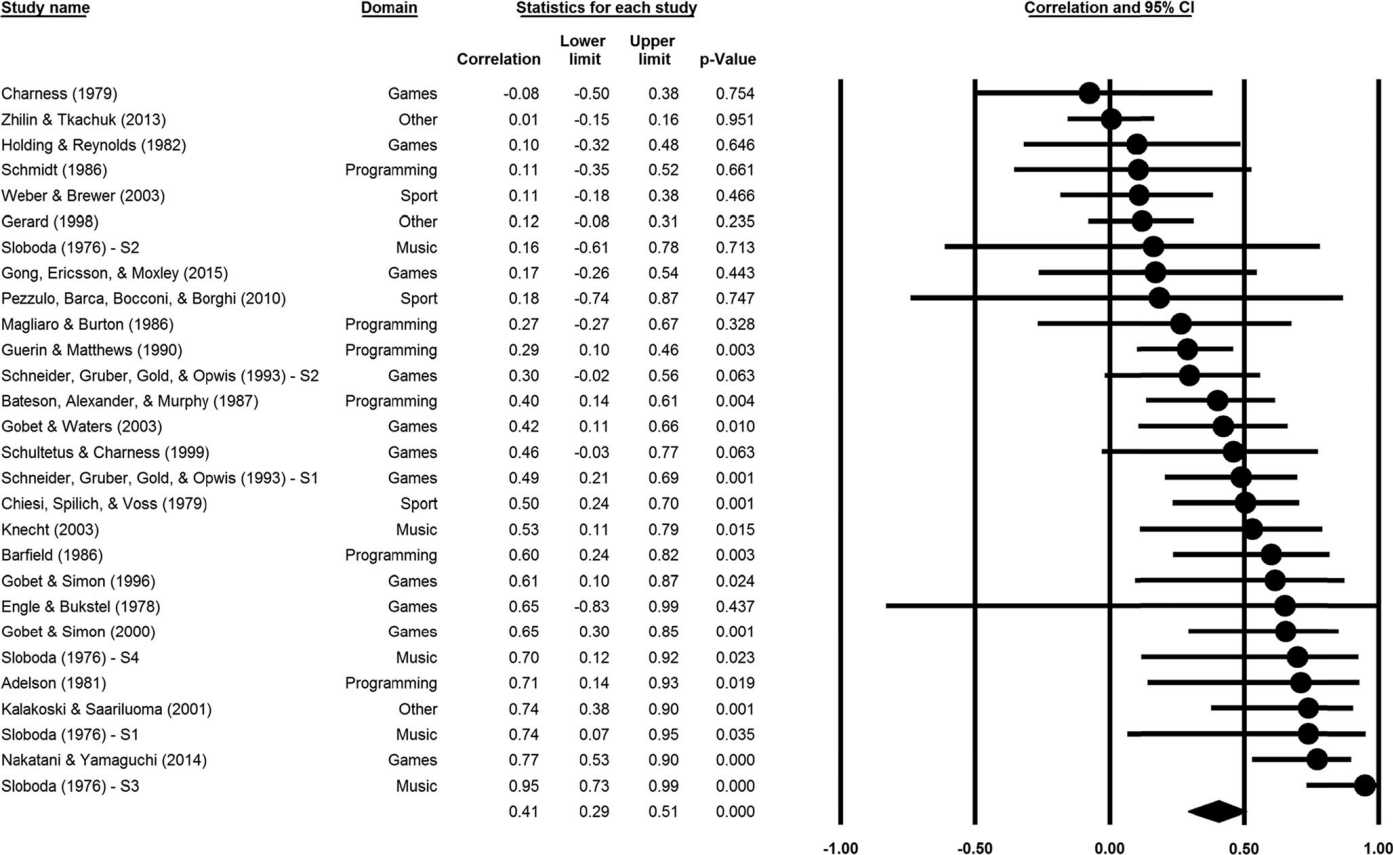
Overall correlation ($\overline{r}$) for skill effect in recalling random material. Correlation coefficients (*circles*) and 95 % CIs (*lines*) are displayed for all effects entered into the meta-analysis. The *diamond at bottom* represents the meta-analytically weighted correlation coefficient ($\overline{r}$). For studies with multiple independent samples, the result of each sample (S1, S2, etc.) is reported separately

A random model (*k* = 28) was built to calculate the overall correlation. The overall correlation was $\overline{r}$ = .41, 95 % CI [.29; .51], *p* < .001 (see Fig. 2). The degree of heterogeneity between effect sizes was *I* ^2^ = 63.06, suggesting potential moderator effects.

#### Moderator analyses

We ran a moderator analysis to evaluate Domain as potential moderator. Domain was a marginally significant moderator, *Q*(4) = 8.69, *p* = .069, *k* = 28. The correlations were $\overline{r}$ = .42, 95 % CI [.25, .56], *p* < .001, *k* = 11, for games; $\overline{r}$ = .69, 95 % CI [.35, .86], *p* < .001, *k* = 5, for music; $\overline{r}$ = .36, 95 % CI [.21, .49], *p* < .001, *k* = 6, for programming; $\overline{r}$ = .30, 95 % CI [-.04, .58], *p* = .083, *k* = 3, for sports; and $\overline{r}$ = .24, 95 % CI [-.09, .52], *p* = .154, *k* = 3, for other domains. The music-related correlation was slightly superior to the other four overall correlations (*b* = 0.62, *z* = 2.85, *p* = .004).

We also performed a moderator analysis to test whether Time of exposition significantly affected the effect sizes. No significant effect was found, *Q*(1) = 0.89, *p* = .346, *k* = 24.

#### Publication bias

Publication bias occurs when experiments showing weak results are systematically excluded from the literature when the sample sizes are small. To test whether our results were affected by publication bias, we created a funnel plot depicting the relation between Fisher’s *Z* and standard error and performed Duval and Tweedie’s (2000) trim-and-fill analysis.

**Fig. 3:**
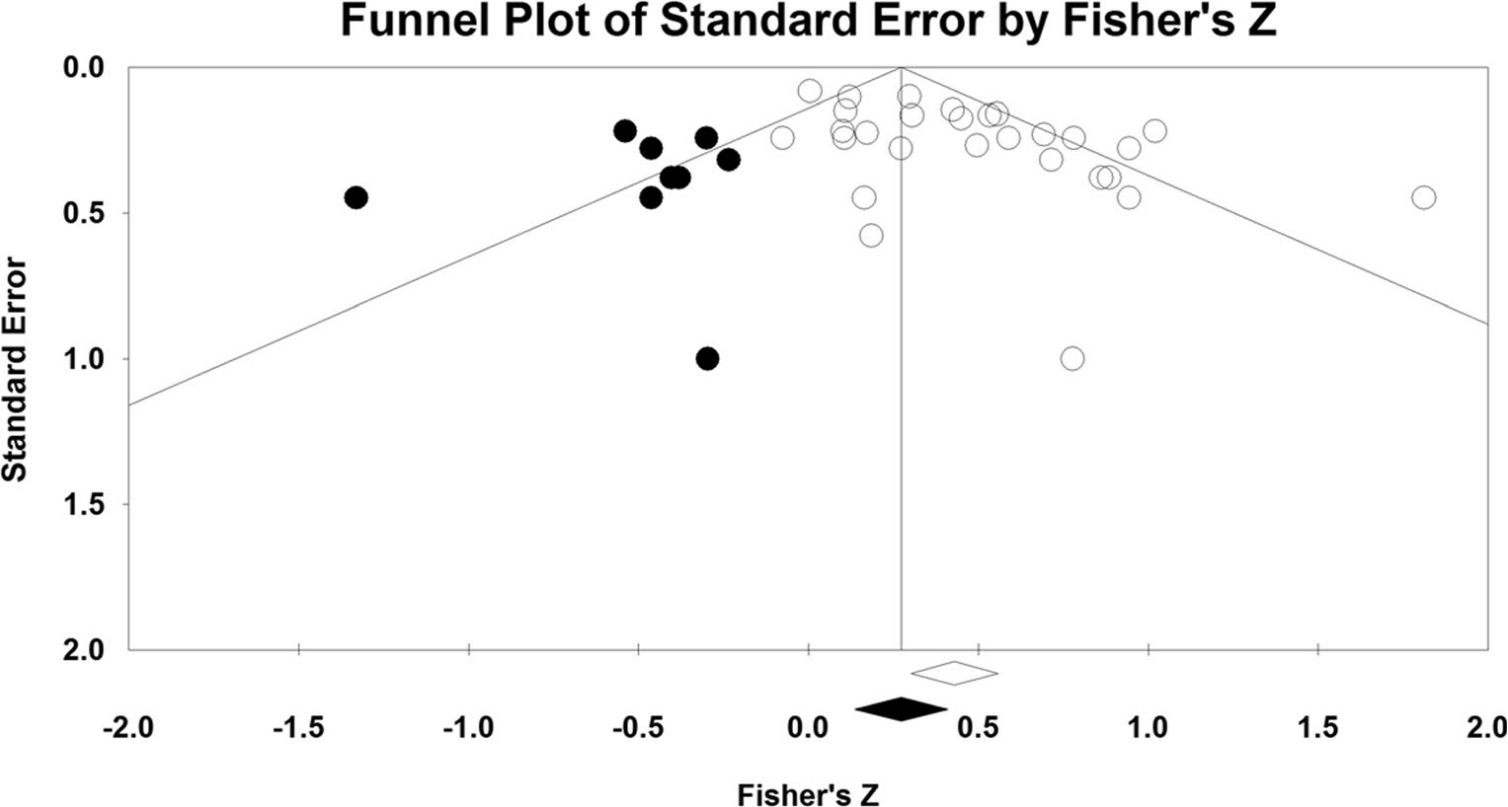
Funnel plot of standard errors and effect sizes (Fischer’s Z). The *white circles* represent the studies included in the meta-analysis and the *black circles* represent the filled-in studies. The *white diamond* indicates the overall correlation estimated from the studies included in the meta-analysis, and the *black diamond* indicates the overall correlation estimated by the trim-and-fill analysis

The funnel plot depicting the relationship between Standard Error and Fisher’s *Z* value looked asymmetrical. The trim-and-fill analysis showed the presence of publication bias. Eight studies were trimmed and the estimate overall correlation was $\overline{r}$ = .29, 95 % CI [.17, .41]. The funnel plot including both the studies in this meta-analysis and the filled in ones is shown in Fig. 3. Finally, the fail-safe *N*—that is, the number of missing studies with effect equal to zero necessary to make the observed effect ($\overline{r}$ = .41) nonsignificant (*p* > .05)—was calculated, and found to be 745.

#### Sensitivity analysis

Two studies included in the meta-analysis presented some methodological issues. As mentioned earlier, Sloboda’s (1976) first experiment included an unspecified number of participants with no music training in the novice group. This condition partly violates one of the inclusion criteria. Also, in Knecht (2003), the novice group did not correctly recall any item (i.e., mean = 0). Although this condition did not violate any of the inclusion criteria, such an unusually poor performance in the novice group might have inflated the effect size.

A sensitivity analysis was thus performed to test the robustness of the results by excluding the two effect sizes. A random model (*k* = 26) was built to calculate the overall correlation. The overall correlation was $\overline{r}$ = .39, 95 % CI [.28, .50], *p* < .001, *I* ^2^ = 64.15. Regarding publication bias, the point estimate was $\overline{r}$ = .28, 95 % CI [.15, .40], with seven effect missing sizes filled in left of the mean.

No significant effect was found for either of the two moderators (*p* = .116 and *p* = .420 for Domain and Time of exposition, respectively). The music-related correlation was still slightly superior to the other four overall correlations (*b* = 0.74, *z* = 2.56, *p* = .010).

### Binomial distribution analysis

The 45 studies included 55 experiments; in 49 cases, the experts outperformed the novices (for a list of the articles, see Table 2 in Appendix A and Appendix B). Assuming a binomial distribution with a probability of success (i.e., experts performing better than the novices) of .50, *n* = 55, and *k* = 49, the probability of obtaining at least 49 successes out of 55 is *p* = 9.11 × 10^-10^.

## Discussion

The results presented in our meta-analysis suggest that experts keep an advantage even when they recall random material; this skill effect is not limited to one specific domain (e.g., chess), but is common to nearly every kind of material, with only sports and “other domains” failing to reach statistical significance. In addition, the overall correlation was significant but no more than moderate.^4^ This outcome corroborates the hypothesis according to which human memory mechanisms are in part based on *small* memory structures (such as chunks), which are stored in LTM (Chase & Simon, 1973; Gobet & Simon, 2000). Experts—who have access to many more of these structures than do novices—are more likely to recognize the patterns that accidentally emerge after domain-specific material is randomized. As previously mentioned, theories of expert memory focusing on high-level structures or holistic processing of stimuli (e.g. Holding & Pfau, 1985; Dreyfus & Dreyfus, 1986) cannot explain this result, because the structures they postulate cannot be used with random stimuli.

### Moderator effects

The moderator analysis showed that the skill effect was more than moderate only with musicians ($\overline{r}$ = .69). This seems to be an empirical anomaly. As suggested by Gobet and Waters (2003) and Knecht (2003), the skill effect is inversely related to the degree of randomness of the material to recall, and it is reasonable to assume that music-related materials used in recall tasks had a lower degree of randomness. For example, the task used in Sloboda’s (1976) experiments consisted of recalling only five notes presented inside a musical stave, with nine possible positions (five lines and four spaces) for every note. Therefore, the number of possible combinations that could have been obtained by randomizing those musical notes was far lesser than—for instance—random chess positions, which usually contained 20–25 pieces placed on 64 possible squares. Thus, the greater skill effect in the domain of music was probably due to the low degree of randomness of the material used in the studies included in the meta-analysis, and not to some other feature specific to the field of music.

Finally, the time of exposition of the stimuli exerted no significant influence on the effect sizes. This outcome suggests that no other memory mechanism—such as encoding new chunks or using semantics—was uniquely used by the experts during the recall of the unstructured material. It is likely that additional time allows both novices and experts to learn new long-term memory chunks (e.g., Gobet & Simon, 2000). Consistent with this hypothesis, the effect was stronger in musicians ($\overline{r}$ = .69) and games players ($\overline{r}$ = .42)—who were exposed just for a few seconds to the stimuli—than in programmers ($\overline{r}$ = .36)—who had up to 10 minutes.

It is worth noting that the lack of effect for the presentation time moderator is different from what Gobet and Simon (2000) found: with random positions, the slope of recall increase was slightly larger for masters than for candidate masters and Class A players, a result that was accurately simulated by their computer model. However, the differences were small, as indicated by the parameter *c* in their Tables 1 and 3. Whether this result generalizes to other domains should be investigated in further experiments, where the presentation time of the stimuli is systematically varied. In the current meta-analysis, the presentation time is confounded with domain.

### Limitations of the study

The present meta-analysis has four limitations that merit discussion. First, the total number of studies (*N* = 24) and participants (*N* = 903) was relatively small. As a consequence, it was not possible to carry out moderator analyses on variables such as age, gender, or expertise level. However, we note that the skill effect was present in nearly all the studies excluded for not providing enough data to calculate an effect size. Those studies often reported not only that randomization reduced the skill effect in the recall task but also that experts kept a small advantage over novices when recalling random material, which is in line with our main analysis.

Second, and linked to the first limitation, the presence of publication bias suggests that the overall correlation we calculated ($\overline{r}$ = .41) is probably an overestimation. Nonetheless, the value estimated by the trim-and-fill analysis ($\overline{r}$ = .29) is still statistically significant, and both values suggest that the skill effect in recalling random material is significant, but at best moderate, a result consistent with the chunking hypothesis. Moreover, the high number (*N* = 745) of studies estimated by the fail-safe analysis and the low probability (*p* = 9.11 × 10^-10^) estimated by the binomial analysis suggest that the skill effect we found is a genuine result.

Third, the randomization methods varied from domain to domain, most likely a necessity as they depend on the structure of a specific domain. In addition, different methods can be used in a single domain. Although this weakness was unavoidable, further research should systematically investigate different methods of randomization in a domain, testing the predictions of a formal model. For example, most studies on chess memory followed Chase and Simon’s method, where pieces from a game position are randomly reassigned to a new square. Gobet and Waters (2003) and Waters and Gobet (2008) explored different methods, including selecting pieces with the same probability, and used the empirical data to test the prediction of CHREST, a chunked-based model.

Finally, we could not correct for measurement error because only five studies provided reliability coefficients for the recall tasks. Moreover, among the domains considered in our meta-analysis, only chess (to the best of our knowledge) uses a rating system (Elo, 1978), whose reliability coefficient has been calculated (*r* = .91; Hambrick et al., 2014). In any case, this limitation does not invalidate the main outcome of the meta-analysis, which is that a moderate skill effect in the recall task still remains even with random material, and that this phenomenon applies to nearly every domain considered.

### Conclusions

The results presented in this meta-analysis show that a skill effect occurs in recall tasks even when the domain-specific material to recall is unstructured. This outcome lends support to the hypothesis according to which human memory mechanisms are in part based on small memory structures such as chunks. Larger, schema-like structures are gradually built on chunks as a function of the exposure to frequent objects and scenes in the environment (Gobet & Chassy, 2009; Gobet & Simon, 1996c). Conversely, theories of expert memory based only on high-level knowledge structures such as schemata or holistic processing cannot explain a skill effect in recalling random material.

One possible alternative explanation is that experts try to recall more items (e.g., chess pieces, music notes) than do novices. To test this hypothesis, Charness and Schultetus (1999) analyzed the performance of chess players in the recall task and controlled for errors of commissions (pieces placed incorrectly). The results showed that the expert chess players (i.e., Elo rating >1999) still outperformed the group of novices.

Another alternative explanation is that experts outperform novices because of their superior working memory (WM; Meinz & Hambrick, 2010). Because individuals whose WM capacity is greater are more likely to acquire expertise in their field, the skill effect we observed might be due to experts’ superior ability to retain elements in WM, and not necessarily to experts’ vaster amount of chunks stored in their LTM. Although further research is needed to test this and other alternative explanations, the greater average age of the experts compared to novices in many of the reviewed studies (e.g., Barfield, 1986; Guerin & Matthews, 1990; Kalakoski & Saariluoma, 2001; Knecht, 2003; Sloboda, 1976) militates against it. The acquisition of expertise is a relatively long process, and thus experts tend to be older than novices (Lehman, 1953). Because WM efficiency decreases as a function of age (Birren & Schaie, 1996), it is unlikely that experts’ advantage at recalling random material is only due to WM ability. Consistent with this hypothesis, a recent meta-analysis (Moxley & Charness, 2013) has shown that performance on the recall of chess positions is negatively associated with age, but positively associated with chess skill. Therefore, experts’ superior ability to recognize small chunks occurring by chance in random material is the most likely explanation.

## Appendix A

**Table 2:** The 45 studies (55 samples) included in the binomial distribution analysis

Study	*N*	Domain	Included in the meta-analysis	Skill effect
Abernethy, Neal, and Konig (1994)	29	Sports (snooker)	No	No
Adelson (1981)	10	Programming	Yes	Yes
Barfield (1986)	22	Programming	Yes	Yes
Bateson, Alexander, and Murphy (1987)	50	Programming	Yes	Yes
Beal (1985)	20	Music	No	Yes
Charness (1979)	20	Games (bridge)	Yes	No
Chase and Simon (1973)	3	Games (chess)	No	No
Chiesi, Spilich, and Voss (1979)	42	Sports (baseball)	Yes	Yes
Cole (1991)	38	Sports (basket)	No	Yes
Coughlin and Patel (1987)	32	Other (medicine)	No	Yes
Egan and Schwartz (1979)	12	Other (symbolic drawings)	Yes	No
Engle and Bukstel (1978)	4	Games (bridge)	Yes	Yes
Frey and Adesman (1976)	13	Games (chess)	No	Yes
Garland and Barry (1991)	80	Sports (football)	No	Yes
Gerard (1998)	100	Other (REA diagrams)	Yes	Yes
Gobet and Clarkson (2004)	12	Games (chess)	No	Yes
Gobet and Simon (1996a)	25	Games (chess)	No	Yes
Gobet and Simon (1996b)	13	Games (chess)	Yes	Yes
Gobet and Simon (2000)	20	Games (chess)	Yes	Yes
Gobet and Waters (2003)	36	Games (chess)	Yes	Yes
Gold and Opwis (1992)	40	Games (chess)	No	Yes
Gong, Ericsson, and Moxley (2015)	23	Games (chess)	Yes	Yes
Guerin and Matthews (1990)	104	Programming	Yes	Yes
Hodge (1997)	20	Sports (martial arts)	No	Yes
Holding and Reynolds (1982)	24	Games (chess)	Yes	Yes
Knecht (2003)	20	Music	Yes	Yes
Lories (1987)	19	Games (chess)	No	Yes
Magliaro and Burton (1986)	16	Programming	Yes	Yes
Nakatani and Yamaguchi (2014)	24	Games (shogi)	Yes	Yes
Norman, Brooks, and Allen (1989)	11	Other (medicine)	No	No
Pezzulo, Borghi, Barca, and Bocconi (2010)	6	Sports (climbing)	Yes	Yes
Reitman (1976)	2	Games (go)	No	Yes
Saariluoma (1984) (Exp. 3)	4	Games (chess)	No	Yes
Saariluoma (1984) (Exp. 4)	4	Games (chess)	No	Yes
Saariluoma (1985)	9	Games (chess)	No	Yes
Saariluoma (1989) (Exp. 1)	8	Games (chess)	No	Yes
Saariluoma (1989) (Exp. 2)	6	Games (chess)	No	Yes
Saariluoma (1989) (Exp. 3)	12	Games (chess)	No	Yes
Saariluoma (1994) (Exp. 1)	12	Games (chess)	No	Yes
Saariluoma (1994) (Exp. 2)	9	Games (chess)	No	Yes
Saariluoma (1994) (Exp. 3)	8	Games (chess)	No	Yes
Saariluoma (1994) (Exp. 4)	10	Games (chess)	No	Yes
Schmidt (1986)	20	Programming	Yes	Yes
Schneider, Gruber, Gold, and Opwis (1993) (Exp. 1)	40	Games (chess)	Yes	Yes
Schneider, Gruber, Gold, and Opwis (1993) (Exp. 2)	40	Games (chess)	Yes	Yes
Schultetus and Charness (1999)	17	Games (chess)	Yes	Yes
Sloboda (1976) (Exp. 1)	8	Music	Yes	Yes
Sloboda (1976) (Exp. 2)	8	Music	Yes	Yes
Sloboda (1976) (Exp. 3)	10	Music	Yes	Yes
Sloboda (1976) (Exp. 4)	10	Music	Yes	Yes
Starkes (1987)	43	Sports (hockey)	No	Yes
Starkes, Caicco, Boutilier, and Sevesk (1990)	17	Sports (dance)	No	Yes
Starkes, Deakin, Lindley, and Crisp (1987)	16	Sports (dance)	No	No
Weber and Brewer (2003)	48	Sports (hockey)	Yes	Yes
Zhilin and Tkachuk (2013)	152	Other (chemistry)	Yes	Yes

## Appendix B

### List of the studies included in the binomial distribution analysis

Abernethy, B., Neal, R. J., & Konig, P. (1994). Visual-perceptual and cognitive differences between expert, intermediate and novice snooker players. *Applied Cognitive Psychology, 18*, 185–211.

Adelson, B. (1981). Problem solving and the development of abstract categories in programming languages. *Memory & Cognition, 9*, 422–433.

Barfield, W. (1986). Expert-novice difference for software: Implications for problem solving and knowledge acquisition. *Behaviour and Information Technology, 5*, 15–29.

Bateson, A. G., Alexander, R. A., & Murphy, M. D. (1987). Cognitive processing differences between novice and expert computers programmers. *International Journal of Man-Machine Studies, 26*, 649–660.

Beal, A. L. (1985). The skill of recognizing musical structures. *Memory & Cognition, 13*, 405–412.

Charness, N. (1979). Components of skill in bridge. *Canadian Journal of Psychology, 33*, 1–16.

Chase, W. G., & Simon, H. A. (1973). Perception in chess. *Cognitive Psychology, 4*, 55–81.

Chiesi, H. L., Spilich, G. J., & Voss, J. F. (1979). Acquisition of domain-related information in relation to high and low domain knowledge. *Journal of Verbal Learning and Verbal Behavior, 18*, 257–273.

Cole, K. C. (1991). *Effects of code interference on the recall of performance-related information* (Doctoral dissertation). Los Angeles: University of Southern California.

Coughlin, L. D., & Patel, V. L. (1987). Processing of critical information by physicians and medical students. *Journal of Medical Education, 62*, 818–828.

Egan, D. E., & Schwartz, E. J. (1979). Chunking in recall of symbolic drawings. *Memory & Cognition, 7*, 149–158.

Engle, R. W., & Bukstel, L. (1978). Memory processes among bridge players of differing expertise. *American Journal of Psychology, 91*, 673–689.

Frey, P. W., & Adesman, P. (1976). Recall memory for visually presented chess positions. *Memory & Cognition, 4*, 541–547.

Garland, D. J., & Barry, J. R. (1991). Cognitive advantage in sport: The nature of perceptual structures. *American Journal of Psychology, 104*, 211–228.

Gerard, J. G. (1998). *REA knowledge acquisition and related conceptual database design performance* (Doctoral dissertation). East Lansing: Michigan State University.

Gobet, F., & Clarkson, G. (2004). Chunks in expert memory: Evidence for the magical number four . . . or is it two? *Memory, 12*, 732–747.

Gobet, F., & Simon, H. A. (1996a). Recall of random and distorted positions. Implications for the theory of expertise. *Memory & Cognition, 24*, 493–503.

Gobet, F., & Simon, H. A. (1996b). Templates in chess memory: A mechanism for recalling several boards. *Cognitive Psychology, 31*, 1–40.

Gobet, F., & Simon, H. A. (2000). Five seconds or sixty? Presentation time in expert memory. *Cognitive Science, 24*, 651–682.

Gobet, F., & Waters, A. J. (2003). The role of constraints in expert memory. *Journal of Experimental Psychology: Learning, Memory, and Cognition, 29*, 1082–1094.

Gold, A., & Opwis, K. (1992). Methoden zur empirischen Analyse von Chunks beim Reproduzieren von Schachstellungen [Methods for the empirical analysis of chunks in recalling chess positions]. *Sprache & Kognition, 11*, 1–13.

Gong, Y., Ericsson, K. A., & Moxley, J. H. (2015). Recall of briefly presented chess positions and its relation to chess skill. *PLOS ONE, 10*, e0118756.

Guerin, B., & Matthews, A. (1990). The effects of semantic complexity on expert and novice computer program recall and comprehension. *The Journal of General Psychology, 117*, 379–389.

Hodge, T. Y. (1997). *Deliberate practice and expertise in the martial arts: The role of context in motor recall* (Doctoral thesis, Queen’s University, Kingston, ON, Canada).

Holding, D. H., & Reynolds, R. I. (1982). Recall or evaluation of chess positions as determinants of chess skill. *Memory & Cognition, 10*, 237–242.

Knecht, M. G. (2003). Music expertise and memory: The relationship between music expertise and memory of music patterns, within various degrees of contextual constraint. *Music Education Research, 5*, 227–242.

Lories, G. (1987). Recall of random and non random chess positions in strong and weak chess players. *Psychologica Belgica, 27*, 153–159.

Magliaro, S., & Burton, J. K. (1986). *Adolescents’ chunking of computer programs*. Paper presented at the Annual Meeting of the American Educational Research Association, San Francisco, CA.

Nakatani, H., & Yamaguchi, Y. (2014). Quick concurrent responses to global and local cognitive information underlie intuitive understanding in board-game experts. *Scientific Reports, 4*. doi:10.1038/srep05894

Norman, G. R., Brooks, L. R., & Allen, S. W. (1989). Recall by expert medical practitioners and novices as a record of processing attention. *Journal of Experimental Psychology: Learning, Memory, and Cognition, 15*, 1166–1174.

Pezzulo, G., Borghi, A. M., Barca, L., & Bocconi, A. L. (2010). When affordances climb into your mind: Advantages of motor simulation in a memory task performed by novice and expert rock climbers. *Brain and Cognition, 73*, 68–73. doi:10.1016/j.bandc.2010.03.002

Reitman, J. S. (1976). Skilled perception in go: Deducing memory structures from inter-response times. *Cognitive Psychology, 8*, 336–356.

Saariluoma, P. (1984). *Coding problem spaces in chess: A psychological study*. Turku, Finland: Societas Scientiarum Fennica.

Saariluoma, P. (1985). Chess players’ intake of task-relevant cues. *Memory & Cognition, 13*, 385–391.

Saariluoma, P. (1989). Chess players’ recall of auditorily presented chess positions. *Experimental Journal of Cognitive Psychology, 1*, 309–320.

Saariluoma, P. (1994). Location coding in chess. *The Quarterly Journal of Experimental Psychology, 47*, 607–630.

Schmidt, A. L. (1986). Effects of experience and comprehension on reading time and memory for computer programs. *International Journal of Man-Machine Studies, 25*, 399–409.

Schneider, W., Gruber, H., Gold, A., & Opwis, K. (1993). Chess expertise and memory for chess positions in children and adults. *Journal of Experimental Child Psychology, 56*, 328–349.

Schultetus, R. S., & Charness, N. (1999). Recall or evaluation of chess positions revisited: The relationship between memory and evaluation in chess skill. *American Journal of Psychology, 112*, 555–569.

Sloboda, J. A. (1976). Visual perception of musical notation: Registering pitch symbols in memory. *Quarterly Journal of Experimental Psychology, 28*, 1–16.

Starkes, J. L. (1987). Skill in field hockey: The nature of the cognitive advantage. *Journal of Sport Psychology, 9*, 146–160.

Starkes, J. L., Caicco, M., Boutilier, C., & Sevesk, B. (1990). Motor recall of experts for structured and unstructured sequences in creative modern dance. *Journal of Sport & Exercise Psychology, 12*, 317–21.

Starkes, J. L., Deakin, J. M., Lindley, S., & Crisp, F. (1987). Motor versus verbal recall of ballet sequences by young expert dancers. *Journal of Sport Psychology, 9*, 222–230.

Weber, N., & Brewer, N. (2003). Expert memory: The interaction of stimulus structure, attention, and expertise. *Applied Cognitive Psychology, 17*, 295–308.

Zhilin, D. M. T., & Tkachuk, L. E. (2013). Chunking in chemistry. *Eurasian Journal of Physics and Chemistry Education, 5*, 39–56.

## Footnotes

1 In Sloboda’s (1976) first experiment, the novice group consisted of four undergraduates with little or no musical training. However, the material consisted of cards with dots drawn on five lines (representing the musical stave). Thus, it is reasonable to suppose that such stimuli were also familiar to those participants who had no experience of music reading.

2 Some study reported the results of several trials. In these cases, we used the data from the first trial or, if no separate results were provided, the participants’ overall average. This procedure was adopted to rule out, where possible, potential confounds such as training and testing effects, and to not violate the statistical independence of the samples.

3 As suggested by Schmidt and Hunter (2015), when the sample was less than 20 participants, Cohen’s *d*s were corrected for upward bias (Hedges & Olkin, 1985) and then converted into point biserial correlations.

4 The effect sizes for meaningful domain-specific material are about 40% to 50% greater than the overall effect sizes reported for unstructured material. For example, the point-biserial correlation for structured material is *r* = .87 in Knecht (2003), and *r* = 0.61 in Gobet and Simon (2000).
